# 

**Published:** 1865-12

**Authors:** 


### OBITUARY RECORD.

Died, at New Orleans, on the 8th of September last, J. L. RIDDLE, M. D., Professor of Chemistry in the University of Louisiana.

Died, in Paris, Oct., 1865, of cerebral apoplexy, Prof. J. F. MALGAIGNE, one of the most eminent, learned and eloquent surgeons and teachers of the French capital.

Died, in London, Nov. 2d, age 66, JOHN LINDLEY, M. D., F. R. S., Professor of Botany at University College, London.

Died, in Paris, of cholera, Dr. BREARD, aged 64 years.

